# A High-Sensitivity Inkjet-Printed Flexible Resonator for Monitoring Dielectric Changes in Meat

**DOI:** 10.3390/s25051338

**Published:** 2025-02-22

**Authors:** Jamal Abounasr, Mariam El Gharbi, Raúl Fernández García, Ignacio Gil

**Affiliations:** Department of Electronic Engineering, Universitat Politècnica de Catalunya, 08222 Terrassa, Spain; mariam.el.gharbi2@upc.edu (M.E.G.); raul.fernandez-garcia@upc.edu (R.F.G.); ignasi.gil@upc.edu (I.G.)

**Keywords:** flexible loop antenna, inkjet-printed sensor, microwave resonance, meat freshness monitoring, dielectric property analysis, real-time food quality assessment, permittivity-based sensing, non-invasive monitoring, 2.4 GHz ISM band, printed electronics

## Abstract

This paper introduces a flexible loop antenna-based sensor optimized for real-time monitoring of meat quality by detecting changes in dielectric properties over a six-day storage period. Operating within the 2.4 GHz ISM band, the sensor is designed using CST Microwave Studio 2024 to deliver high sensitivity and accuracy. The sensing mechanism leverages resonance frequency shifts caused by variations in permittivity as the meat degrades. Experimental validation across five samples showed a consistent frequency shift from 2.14 GHz (Day 0) to 1.29 GHz (Day 5), with an average sensitivity of 0.173GHz/day. A strong correlation was observed between measured and simulated results, as evidenced by linear regression ($R^{2}=0.984$ and $R^{2}=0.974$ for measured and simulated data, respectively). The sensor demonstrated high precision and repeatability, validated by low standard deviations and minimal frequency deviations. Compact, printable, and cost-effective, the proposed sensor offers a scalable solution for food quality monitoring. Its robust performance highlights its potential for integration into IoT platforms and extension to other perishable food products, advancing real-time, non-invasive, RF-based food safety technologies.

## 1. Introduction

Food safety and quality are critical aspects of global public health and food security. The increasing complexity of food supply chains, coupled with growing consumer demand for fresh, high-quality products, requires robust monitoring systems [1]. Ensuring that food, especially perishable items such as meat, remains safe and of high quality throughout its storage and distribution is essential to preventing foodborne illness, reducing economic losses, and building consumer confidence [2,3]. Contaminated or substandard food creates serious health risks to consumers leading to illnesses associated with ingestion of food, which include acute bowel disorders, chronic illness, and mortality [4]. It is reported by the World Health Organization (WHO) that millions of people across the globe suffer from foodborne diseases every year with, contamination by pathogenic organisms such as Salmonella, Listeria, and E. coli as key factors. It is also important to realize that the spoilage of meat leads to the generation of poisonous by-products as well as the growth of pathogens, thus warranting the need for stringent supervision [5]. On a worldwide scale, food safety and food quality are important issues of food security. Spoilage and waste appear as a challenge to food security, especially in underdeveloped countries with very little cold chain facilities. A developed means of supervision may help to manage food waste by tracing the most susceptible products dressed to be lost in the chain, which will help to take proper measures as early as possible.

Technological advances in radio frequency (RF) and microwave technologies transform food safety and quality assurance by providing real-time, non-invasive monitoring solutions [6]. These techniques leverage the interaction between electromagnetic waves and food materials to detect certain key quality parameters such as spoilage. RF-based systems, including antenna-based sensors and RFID sensors, allow for continuous monitoring of environmental conditions and dielectric property changes in food [7]. By offering a sophisticated and scalable approach to food monitoring, RF and microwave techniques pave the way for safer and more efficient food quality management. In the context of food safety and quality assurance, dielectric properties, particularly permittivity, serve as reliable indicators of structural and compositional changes in perishable foods [8]. As food ripens or spoils, its chemical composition undergoes significant alterations, leading to measurable changes in its dielectric properties. These changes make permittivity a cornerstone of radio frequency (RF)- and microwave-based sensing and monitoring technologies [9]. By accurately measuring these properties, advanced systems can deliver real-time insights into food freshness, enhancing safety, reducing waste, and ensuring better quality control throughout the supply chain.

A trained individual, such as a store manager, can assess the quality of meat by sight, smell, and touch. However, this approach poses potential health risks to the inspector. In addition, relying solely on subjective judgment leads to the possibility of errors that are not supported by objective data. Furthermore, when touch and smell are used in the inspection process, it becomes impossible to assess the packaged products [10]. The existing meat quality monitoring systems are costly to implement and not widely utilized. Despite their presence, cases of health issues and fatalities resulting from the consumption of spoiled meat are still occur.

To address these challenges, several studies have demonstrated the effectiveness of RF and microwave techniques for monitoring perishable foods such as meat, dairy, and seafood [11,12,13]. These methods offer advantages over traditional approaches, including faster processing times, non-destructive analysis, and improved scalability. For example, a UHF RFID system was used to monitor frozen meat using received signal strength indicator (RSSI) data, as reported in [14]. This method demonstrated monotonic relationships between RSSI values, temperature, and hardness during defrosting, enabling effective cold chain monitoring and safety. Another technique was introduced in [15], where dielectric measurements were used to assess meat freshness by monitoring changes in permittivity and conductivity over storage intervals. Using capacitance and conductance data measured with an LCZ meter across frequencies from 10 kHz to 1 MHz, the study demonstrated a decline in these dielectric properties with increasing storage time. In addition, an IoT-based system combining cameras and air quality sensors, using deep learning models to analyze color changes and predict meat freshness in real time, was reported in [16]. This system requires complex calibration for different types and conditions of meat, and its applicability may be limited to specific use cases. However, all of the studies mentioned above rely primarily on rigid substrates, and these kinds of substrates may not be ideal for applications that require flexibility, such as integration into smart packaging or wearable systems for real-time monitoring.

This paper presents a flexible antenna-based sensor for monitoring meat (beef) freshness over six days using microwave signals. The sensor, a circular loop antenna printed on a flexible polyamide substrate, detects shifts in resonance frequency caused by changes in the meat’s dielectric properties as it degrades. These shifts alter the antenna’s resonance characteristics, enabling non-invasive freshness detection. Measurements were conducted using a Vector Network Analyzer (VNA) to observe resonance frequency shifts over time.

The analysis was limited to six days because the meat was visibly rotten beyond this period, making further investigation unnecessary. Additionally, resonance frequency results on subsequent days showed no significant changes compared to Day 6, confirming complete spoilage, which is evident through visual and sensory inspection. Extending the analysis further was, thus, unnecessary for food quality assessment.

## 2. Sensor Design and Working Principle

### 2.1. Sensor Design and Manufacturing

**Figure 1:**
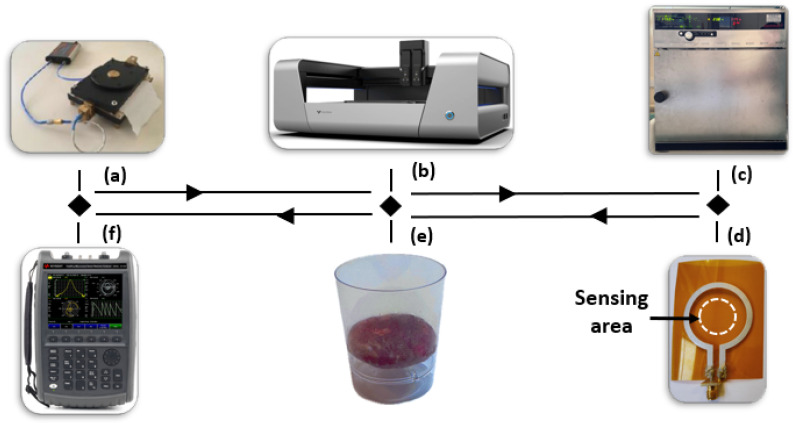
Overview of the experimental setup and fabrication process: (**a**) Q-meter used for extracting electrical properties of the substrate; (**b**) Voltera NOVA inkjet and extrusion printer used for antenna fabrication; (**c**) Memmert oven for drying and curing the printed ink to ensure proper adhesion and distribution; (**d**) final fabricated loop antenna; (**e**) 10 g of meat sample; (**f**) vector network analyzer (VNA) for antenna performance validation.

**Figure 2:**
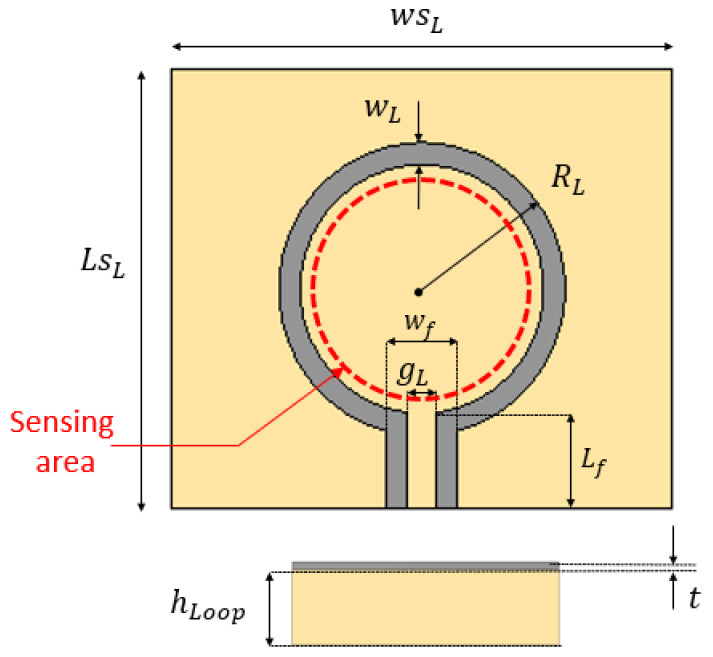
The geometrical parameters of the proposed antenna.

The sensor, based on a loop antenna design, was developed to operate within the 2.4 GHz ISM band and optimized using CST Microwave Studio 2024. The geometrical parameters of the proposed antenna are detailed in Table 1 and illustrated in Figure 1 and Figure 2.

These parameters were tuned to achieve optimal performance and ensure efficient electromagnetic coupling with the target samples. Polyimide (PI) films, commonly known by the brand name Kapton, were selected as the substrate material due to their excellent dielectric properties ($\epsilon_{r}=3.5$, loss tangent = 0.0027), lightweight structure, and durability under extreme environmental conditions. To ensure consistency for high-performance microwave applications, the electrical properties of the substrate were verified using a Q-meter, as shown in Figure 1a.

The conductive traces of the loop antenna were fabricated using an inkjet and extrusion printer (Voltera NOVA, Figure 1b) with a 225 μm nozzle. This system simplifies the calibration process by automating parameters such as dispensing height, ink pressure, and temperature, making it highly user-friendly and efficient. The printing process typically requires less than 15 min to complete. Inkjet printing was chosen for its rapid prototyping capabilities, minimal material waste, and suitability for flexible substrates, making it an ideal technique for disposable and scalable food quality sensors.

After printing, the antennas were dried in a Memmert oven (Figure 1c) at 50 °C for 15 min to ensure proper ink adhesion and uniform distribution. This step is critical for maintaining reliable conductivity and achieving stable sensor performance. The final fabricated loop antenna is shown in Figure 1d, and it demonstrated excellent electrical matching to the standard 50Ω impedance. This performance was validated using a vector network analyzer (VNA, Figure 1f).

To assess its functionality, the sensor was tested with a meat sample (Figure 1e) placed directly on the loop in the sensing area illustrated in Figure 2. Measurements were collected over multiple days to analyze the impact of permittivity variations on the reflection coefficient ($S_{11}$) and resonance frequency behavior. This robust and systematic design ensures the sensor’s reliability for dielectric sensing applications in meat quality evaluation.

### 2.2. Working Principle

The proposed loop antenna sensor operates by generating a localized electromagnetic (EM) field in its near-field region. When a material, such as meat, is placed within this field, the electromagnetic waves interact with the dielectric properties of the material, such as its relative permittivity ($\epsilon_{r}$). This interaction modifies the antenna’s impedance by either absorbing or reflecting the EM energy, resulting in measurable changes in the reflection coefficient ($S_{11}$) and a shift in the resonance frequency ($f_{r}$).

At the resonance frequency, the antenna’s impedance is minimized, enabling strong energy coupling with the surrounding medium. This occurs when the inductive reactance ($X_{L}$) of the loop antenna matches the capacitive reactance ($X_{C}$) of the surrounding system. The two reactances cancel each other out, leaving only the resistive component in the total impedance:

(1) $$Z_{\mathrm{tot}}=R+j\left(X_{L}-X_{C}\right)$$

At resonance, since $X_{L}=X_{C}$, the impedance simplifies to

(2) $$Z_{\mathrm{tot}}=R$$

This purely resistive impedance results in the lowest impedance value, allowing maximum current flow in the loop. The strong current flow enhances the interaction between the antenna and the surrounding material, amplifying the sensor’s sensitivity to dielectric changes. As the dielectric properties of the material affect the EM field distribution, the resonance frequency and reflection coefficient shift accordingly.

**Figure 3:**
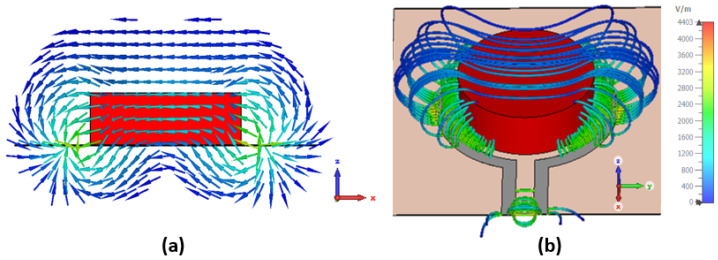
Simulated electric field highlighting the interactions between the loop antenna and the meat sample: (**a**) side view and (**b**) 3D view of the E field.

The interaction between the loop antenna and the material is illustrated in Figure 3, which highlights how the loop antenna generates a localized EM field that interacts with the material. The highest intensity regions, shown in red and orange, are concentrated near the loop antenna edges, while the field weakens radially outward. The meat sample perturbs the field, as evidenced by the redistribution of field lines, confirming the antenna’s sensitivity to dielectric variations in the sample.

The specific composition of the meat, along with the effective dielectric properties used in this study, is detailed in the following subsection. These properties were carefully selected to ensure accurate simulation and analysis of the interaction between the loop antenna and the meat samples.

## 3. Experimental Setup and Measurement Workflow

To investigate the dielectric properties of beef samples and their interaction with the loop antenna, a systematic experimental workflow was implemented. Each sample was carefully prepared to weigh exactly 10 g, with a composition of 80% muscle and 20% fat, reflecting a typical profile for evaluating meat quality. The samples were cylindrical in shape, with a radius of 1.5 cm and a height of 1.5 cm, corresponding to a volume of approximately 10.61 cm^3^. These geometric and compositional parameters were used for both experimental measurements and simulation models to ensure consistency and accuracy in the analysis. Five samples were prepared and tested daily over a six-day period, providing a robust dataset for analysis. The relative permittivity ($\epsilon_{r}$) and loss tangent (tanδ) of the meat were calculated using a weighted average approach based on the dielectric properties of muscle and fat, ensuring accurate modeling and analysis. These properties were derived using the following equations [17,18]:

(3) $$\epsilon_{\mathrm{eff}}=f_{\mathrm{muscle}}\cdot \epsilon_{\mathrm{muscle}}+f_{\mathrm{fat}}\cdot \epsilon_{\mathrm{fat}}$$

(4) $$\tan\delta =\frac{\sigma }{\omega \epsilon_{0}\epsilon_{r}}$$

where the variables are as follows:

- $\epsilon_{\mathrm{eff}}$: effective relative permittivity of the mixture.
- $f_{\mathrm{muscle}}=0.8$ and $f_{\mathrm{fat}}=0.2$: weight fractions of muscle and fat.
- $\epsilon_{\mathrm{muscle}}$ and $\epsilon_{\mathrm{fat}}$: relative permittivities of muscle and fat.
- σ: conductivity of the mixture.
- ω=2πf: angular frequency, where f=2.4GHz.
- $\epsilon_{0}=8.854\times 10^{-12}\;F/m$: permittivity of free space.

Using these equations and the dielectric properties of muscle ($\epsilon_{\mathrm{muscle}}\approx 49,\;\sigma_{\mathrm{muscle}}\approx 1.3\;S/m$) and fat ($\epsilon_{\mathrm{fat}}\approx 5.2,\;\sigma_{\mathrm{fat}}\approx 0.05\;S/m$), the effective properties of the mixture were calculated as follows:

(5) $$\epsilon_{\mathrm{eff}}=\left(0.8\cdot 49\right)+\left(0.2\cdot 5.2\right)\approx 40.96$$

(6) $$\tan\delta =\frac{1.048}{2\pi \cdot 2.4\times 10^{9}\cdot 8.854\times 10^{-12}\cdot 40.96}\approx 0.020$$

These calculated values were used to simulate the electromagnetic interaction between the loop antenna and the meat sample.

The storage conditions were carefully controlled to simulate realistic handling scenarios while minimizing environmental disturbances. Each sample was stored for 12 h at 7 °C (refrigerated) to slow degradation, followed by 8 h at 25 °C (room temperature) to mimic typical exposure conditions. This cycle was repeated daily to reflect practical storage variations. To mitigate the influence of ambient humidity and temperature fluctuations, all measurements were performed immediately after removing the samples from refrigeration, ensuring consistency in dielectric behavior and minimizing external interference.

**Figure 4:**
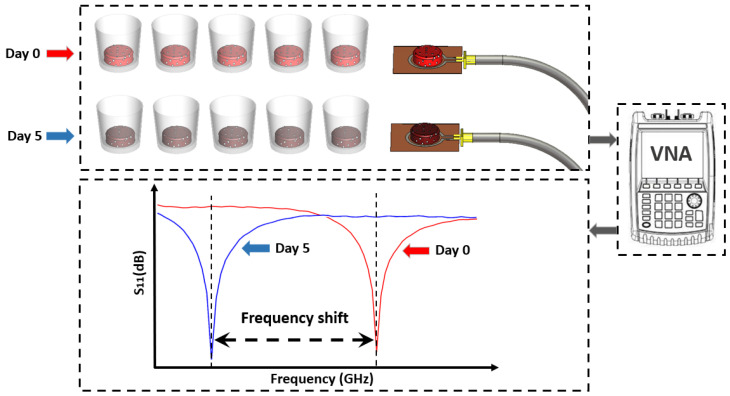
Three-dimensional model representation of the experimental protocol for assessing meat quality using microwave sensing. The model depicts fresh samples (Day 0) and aged samples (Day 5) placed in containers, connected to a microwave sensing setup and a vector network analyzer (VNA).

**Figure 5:**
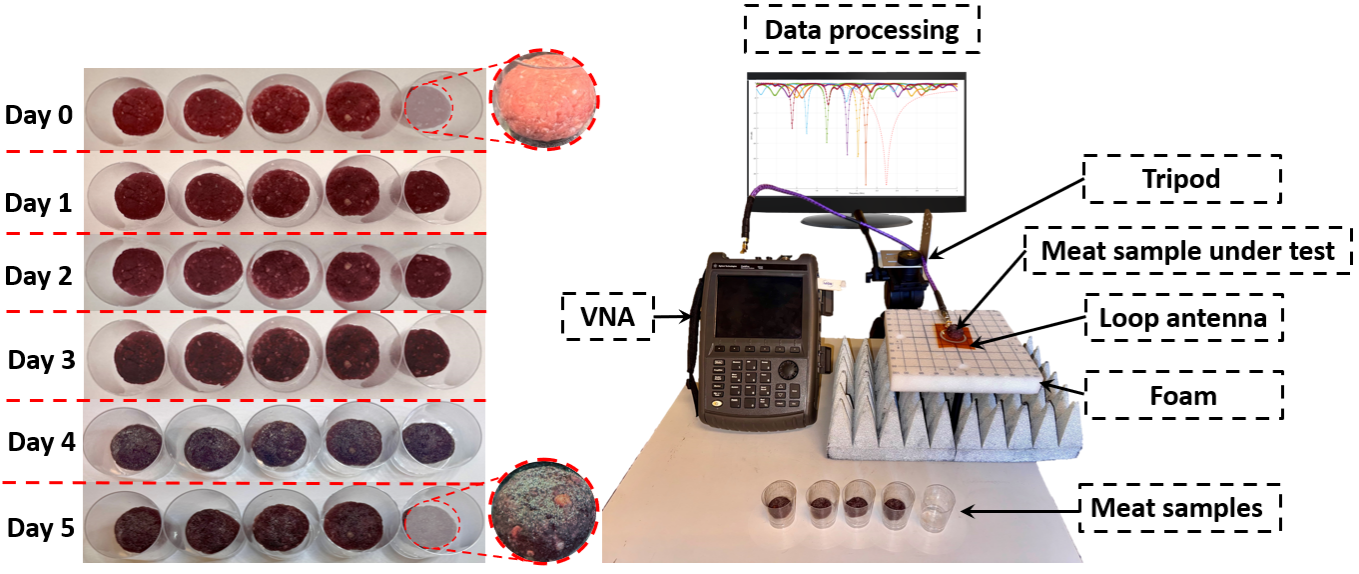
Comprehensive experimental setup for analyzing dielectric property variations in meat samples from Day 0 to Day 5.

The experimental setup included a loop antenna designed to operate within the 2.4 GHz ISM band and a vector network analyzer (VNA) to measure the reflection coefficient ($S_{11}$) over a frequency range of 1–3 GHz, as presented in Figure 4 and Figure 5. Each sample was carefully positioned on the loop antenna to ensure proper coupling with the electromagnetic field. The placement of the sample on the loop antenna allowed direct interaction between the localized electromagnetic field and the dielectric properties of the meat.

For data collection, $S_{11}$ measurements were recorded across the frequency band for each sample. Five samples were measured daily, starting from Day 0 (fresh meat) to Day 5 (stored meat). Measurements were repeated under identical conditions each day to track trends in resonance frequency over time. Data acquisition and analysis were performed using MATLAB 2024B, which facilitated the comparison of $S_{11}$ variations across frequencies and the statistical analysis of all samples.

Data processing involved analyzing the shifts in resonance frequency, identifying changes in the reflection coefficient, and performing statistical comparisons across all days, as described in Figure 4. This comprehensive approach enabled precise monitoring of changes in the dielectric properties of the meat over time, providing valuable insights into the relationship between meat quality and its electromagnetic response.

## 4. Results and Discussion

The experimental results in this section present the capability of the loop antenna to detect variations in the dielectric properties of meat samples stored under controlled conditions, as shown in Figure 5. By monitoring changes in the reflection coefficient ($S_{11}$) and resonance frequency over time, the antenna’s sensitivity to changes in permittivity caused by meat aging, storage, and environmental variations is assessed. The measurements were benchmarked against simulated data to confirm consistency and establish a correlation between experimental and theoretical observations.

Additionally, statistical analyses were performed to evaluate the repeatability of the measurements and quantify the observed frequency shifts resulting from the effects of aging, storage, and environmental factors on the meat samples. The results are discussed with a focus on the sensor’s performance, the influence of material properties on measurement accuracy, and potential applications in food quality assessment.

### 4.1. Antenna Sensor Evaluation in Free Space

**Figure 6:**
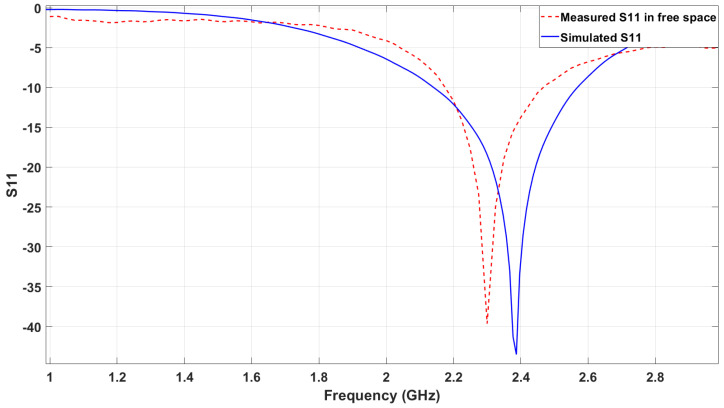
Comparison of measured and simulated $S_{11}$ in free space.

The reflection coefficient ($S_{11}$) of the loop antenna was evaluated both experimentally and through simulation to benchmark the antenna’s performance in free-space conditions. Figure 6 presents a comparative analysis of the measured and the simulated $S_{11}$ as a function of frequency within the 1 GHz to 3 GHz range. The simulated response exhibits a sharp resonance dip at 2.4 GHz, which corresponds to the antenna’s design frequency in the ISM band. The measured response closely follows the simulation, showing a resonance dip at approximately 2.14 GHz. This corresponds to a frequency shift of approximately 10.83%.

The measured response is slightly lower in frequency than the simulated response, which can be attributed to fabrication tolerances and variations in material properties. Factors such as the actual permittivity and thickness of the substrate, which may deviate slightly from simulation inputs, could lead to this shift. Additionally, environmental conditions during measurement might contribute to further deviations.

Despite this shift, the close agreement between the simulated and measured data validates the design process and highlights the effectiveness of the fabrication method. The minimum reflection at resonance confirms good impedance matching to the standard 50Ω system, ensuring efficient energy coupling and minimal power loss at the operational frequency. This benchmark establishes a reliable reference for subsequent experiments involving varying permittivity conditions caused by meat samples.

### 4.2. Permittivity Variation over Days

To validate the hypothesis regarding the influence of permittivity variation on resonance frequency shifts, a parametric sweep simulation was conducted in CST Studio Suite. This simulation utilized a cylindrical meat model with a radius of 1.5 cm, a height of 1.5 cm, and a volume of approximately 10.61 cm^3^, consistent with the experimentally prepared samples. The relative permittivity ($\epsilon_{r}$) of the modeled meat sample was varied, with initial and final values ranging from $\epsilon_{r}=37$ to $\epsilon_{r}=91$, as derived and justified in earlier calculations. The goal of this simulation was to observe and quantify the impact of changing dielectric properties on the resonance frequency behavior of the antenna system.

**Figure 7:**
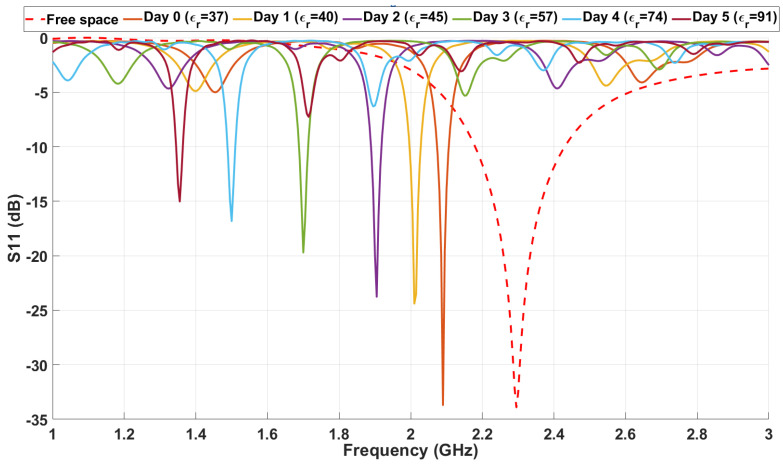
Simulated $S_{11}$ response over the frequency range for various permittivity values corresponding to different days.

Figure 7 illustrates the results of the simulation, showing a clear trend in the frequency response: as the relative permittivity of the material increased, the resonance frequency consistently shifted towards lower values. For the initial permittivity value ($\epsilon_{r}=37$), the simulated resonance frequency was approximately 2.1 GHz, corresponding to a state where electromagnetic coupling with the material was relatively low.

However, as the permittivity increased to its maximum value of $\epsilon_{r}=91$, the resonance frequency decreased significantly to approximately 1.3 GHz. This marked shift reflects the increased energy storage capacity of higher-permittivity materials, which modifies the electromagnetic field distribution and results in a reduced resonance frequency.

**Figure 8:**
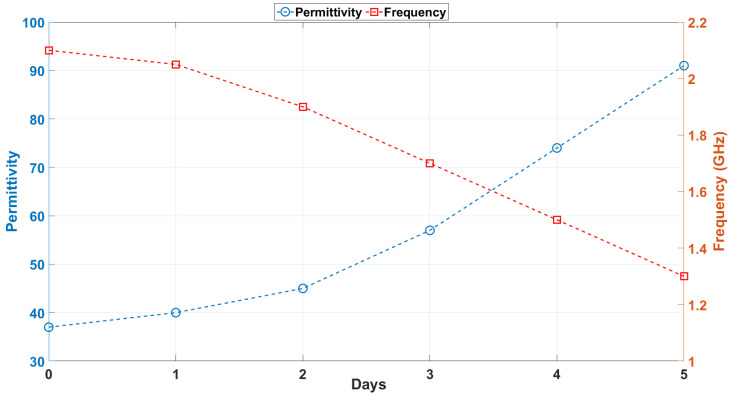
Dynamic relationship between simulated permittivity and frequency across days.

The observed trend presented in Figure 8 aligns closely with theoretical predictions, where materials with higher permittivity are expected to exhibit stronger electromagnetic coupling due to increased polarization. This effect reduces the antenna’s operating frequency as the system’s inductive and capacitive reactance components adjust to the new material properties. Such behavior is indicative of the sensor’s sensitivity to the dielectric properties of the surrounding medium and highlights its potential for detecting subtle variations in material composition.

By performing a comprehensive parametric sweep simulation, the permittivity values were strategically selected to align with the frequencies observed in the measurement results (discussed in later sections). This approach ensures that the simulated frequency shifts correspond closely to the experimentally obtained data, thereby validating the reliability of the proposed sensing mechanism. The consistency between the simulation and measurement outcomes highlights the antenna’s capability to effectively monitor dielectric changes over time.

In addition, the selected permittivity values cover a broad range, from low-loss materials ($\epsilon_{r}=37$) to highly lossy materials ($\epsilon_{r}=91$), reflecting realistic variations in biological tissues. This comprehensive range demonstrates the sensor’s adaptability for diverse applications where dielectric property variations are critical.

### 4.3. Experimental Observations

#### 4.3.1. Average $S_{11}$ Measurements over Six Days

The reflection coefficient ($S_{11}$) was measured daily for five beef samples over a six-day period to investigate how the dielectric properties of the samples evolved during storage and were impacted by environmental conditions. Each measurement covered a frequency range of 1 GHz to 3 GHz, with the goal of identifying shifts in the resonance frequency and overall trends in the reflection behavior. These measurements were averaged across all five samples for each day, providing a generalized representation of the changes in $S_{11}$ over time while minimizing sample-specific variability.

**Figure 9:**
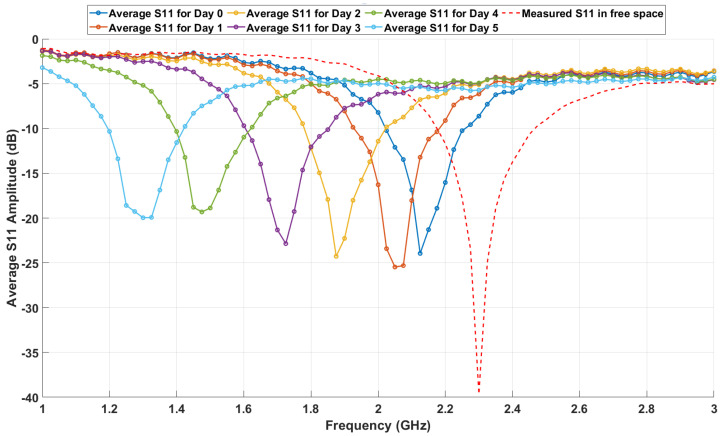
Average ($S_{11}$) measurements over six days.

Figure 9 presents the average $S_{11}$ response for each day, starting from Day 0 (representing fresh samples) to Day 5 (representing aged samples). A distinct downward shift in the resonance frequency is observed over the storage period. On Day 0, the resonance frequency was approximately 2.1 GHz, closely matching the simulated values under initial conditions. By Day 5, the resonance frequency had decreased to approximately 1.3 GHz, reflecting the impact of storage-induced changes on the dielectric properties.

Importantly, the bandwidth remained consistent over the six-day period, indicating that the antenna’s quality factor (Q-factor) and energy coupling characteristics were unaffected. This stability ensures reliable sensitivity to resonance frequency shifts without distortion.

The environmental conditions—specifically, the 8-hour exposure to room temperature (25 °C) after 12 h of refrigerated storage (7 °C)—accelerated the aging process. This is evident from the resonance frequency shifts and increased energy dissipation, as reflected in the broadening and shallowing of the $S_{11}$ dips over time. These changes are attributed to enzymatic activity, microbial growth, and moisture redistribution, which collectively increase the effective permittivity and conductivity of the meat samples.

The free-space $S_{11}$ curve, represented by a red dashed line in Figure 9, serves as a baseline measurement. It represents the loop antenna’s resonance behavior in the absence of any dielectric loading. Compared to the free-space baseline, the curves for Days 0 through 5 exhibit significant shifts in the resonance frequency, highlighting the impact of the meat’s dielectric properties on the antenna’s performance.

These observations confirm the strong correlation between permittivity changes in the meat samples and the measured resonance frequency shifts. The gradual downward frequency trend aligns with theoretical predictions, where materials with higher permittivity values lead to increased energy storage and a corresponding reduction in resonance frequency. Furthermore, the environmental exposure significantly influences this trend by enhancing the dielectric property changes.

It is important to note that the measurements were conducted over six days because, beyond this period, the results began to show a stabilized trend. Measurements taken on Days 7 and 8 showed similar results to Day 6, indicating stabilization in the dielectric behavior of the samples. On day 9, the meat samples had deteriorated to the point of spoilage, making further analysis impractical. This observation underscores the six-day window as critical for assessing changes in meat quality using this sensing approach.

#### 4.3.2. Resonant Frequency Trends over Time

**Figure 10:**
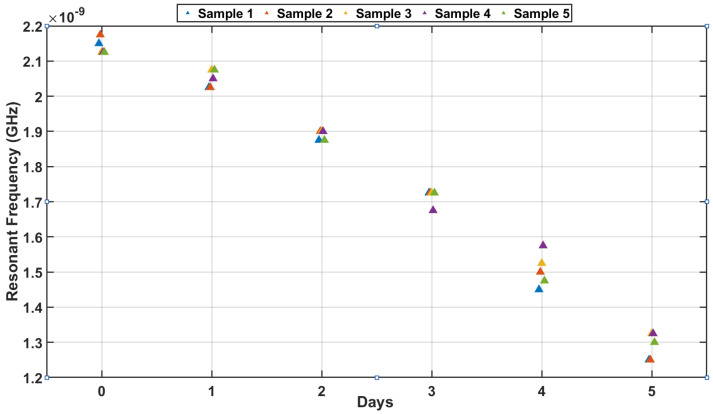
Trend of measured resonance frequencies across individual samples over six days.

To assess the uniformity of the experimental process and the consistency of the prepared meat samples, the resonance frequency was analyzed across five identical samples over a period of six days. Figure 10 illustrates the resonance frequency trend for each sample, demonstrating that despite minor fluctuations, all samples exhibited highly consistent resonance frequency behavior throughout the storage period. This consistency validates the preparation process and ensures that the samples were homogeneous in composition and structure, resulting in nearly identical electromagnetic responses.

The overlapping trends confirm the reliability of the experimental procedure and eliminate concerns of variability due to differences in individual samples. These results highlight the effectiveness of the sensing mechanism in delivering reproducible and precise measurements, reflecting the loop antenna’s sensitivity and reliability in detecting dielectric property changes in meat samples.

#### 4.3.3. $S_{11}$ Amplitude Comparison Across All Samples

**Figure 11:**
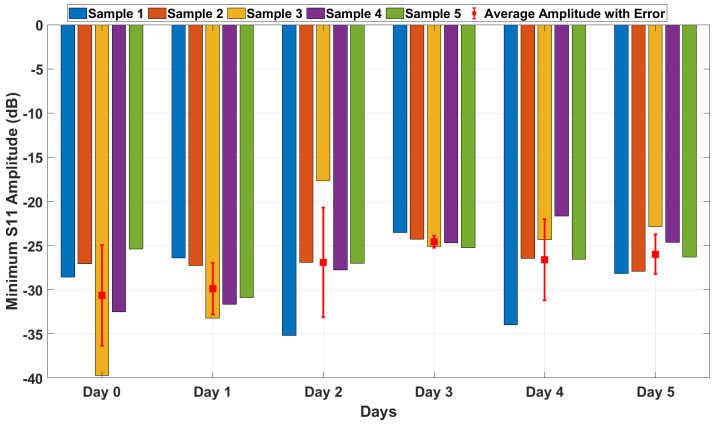
Measured minimum $S_{11}$ amplitudes with average and error bars over days.

The evaluation of the minimum $S_{11}$ amplitude over six days offers valuable insights into the sensitivity and consistency of the proposed antenna-based sensing system. As depicted in Figure 11, the bar plots demonstrate that the minimum $S_{11}$ amplitude values for the five identical meat samples remain consistent throughout the six-day period, with only minor variability observed. This consistency highlights the reliability of the experimental setup and confirms that the prepared samples exhibit comparable dielectric properties, ensuring uniform electromagnetic responses across measurements.

The red squares in Figure 11, representing the average $S_{11}$ amplitude, shows a slight upward trend over the storage period. This trend reflects the impact of meat degradation on the reflection coefficient, which aligns with theoretical expectations. As the meat ages, changes in its dielectric properties, such as increased permittivity and conductivity due to spoilage, result in higher energy absorption. This behavior leads to a gradual reduction in the reflection magnitude, further confirming the sensor’s ability to monitor the dielectric property changes associated with meat spoilage.

The error bars in the figure indicate minimal variability among the samples, underscoring the homogeneity of the meat preparation process and the robustness of the antenna’s sensing mechanism. Notably, the slightly higher variability observed on Day 2 could be attributed to environmental factors or initial sample-specific conditions. These factors, however, are mitigated in subsequent days as the samples degrade more uniformly, resulting in reduced variability. Overall, the observations confirm the antenna’s capability to consistently and accurately detect changes in the dielectric properties of the samples over time.

### 4.4. Simulated and Measured Data Comparison

**Figure 12:**
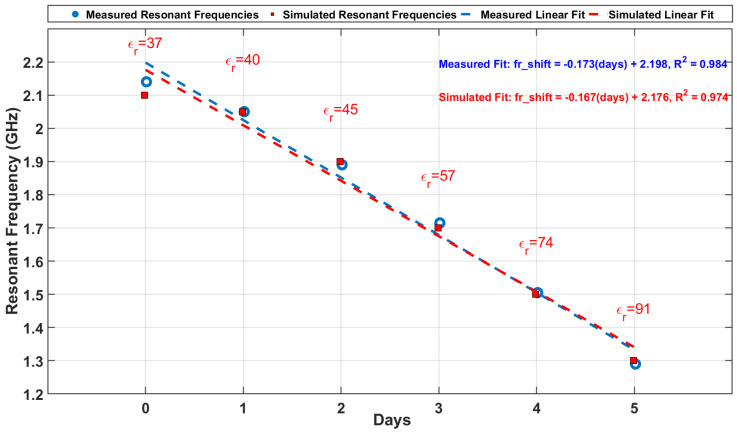
Linear regression analysis of measured and simulated resonant frequencies over time.

Figure 12 presents the comparison between the simulated and measured resonant frequencies for Days 0 through to 5. The simulated frequencies, derived from permittivity values ($\epsilon_{r}$), exhibit strong agreement with the measured data, as illustrated by the close alignment of the points and regression lines. The maximum observed error between the simulated and measured frequencies is 0.04 GHz, confirming the high accuracy of the measurement system.

The standard deviations (σ) of the measured data remain consistently low across all days, as shown in Table 2. The largest standard deviation (0.048GHz) was observed on Day 4, which could be attributed to minor environmental variations or handling differences. However, this variability is well within acceptable limits, demonstrating the robustness of the measurement setup.

The linear regression analysis further validates the correlation between the simulated and measured data, with the regression equations and $R^{2}$ values displayed in Figure 12. These high $R^{2}$ values indicate a very strong linear relationship between the resonant frequency and the number of storage days, reflecting the sensor’s sensitivity to changes in dielectric properties.

The absolute error (Error) between the simulated ($f_{\mathrm{sim}}$) and measured ($f_{\mathrm{meas}}$) resonant frequencies is calculated as

(7) $$\mathrm{Error}=\left|f_{\mathrm{meas}}-f_{\mathrm{sim}}\right|$$

The standard deviation (σ) of the measured resonant frequencies across five samples for a specific day is given by

(8) $$\sigma =\sqrt{\frac{1}{n}\sum_{i=1}^{n}{\left(f_{i}-\bar{f}\right)}^{2}}$$

where

- $f_{i}$ is the resonant frequency of sample *i*,
- $\bar{f}$ is the mean resonant frequency,
- *n* is the number of samples (5 in this study).

The trend of decreasing resonant frequency with increasing permittivity aligns with theoretical expectations. As the meat degrades, spoilage-related changes in its composition lead to an increase in permittivity, causing a downward shift in the resonance frequency. This frequency shift was observed to decrease consistently from 2.14 GHz on Day 0 to 1.29 GHz on Day 5, corresponding to an average sensitivity of 0.173 GHz/day. This behavior underscores the loop antenna’s suitability for real-time, non-invasive monitoring of food quality, while the consistently low standard deviations demonstrate the repeatability and reliability of the system.

#### Comparative Analysis with Existing Technologies

Table 3 summarizes the current sensor technologies for food quality monitoring, comparing their detection methods, measured parameters, target samples, sizes, and fabrication complexities. Existing technologies such as RF energy harvesting, dielectric characterization, VOC detection, and RSSI-based systems are tailored for measuring parameters like color values, temperature, and frequency shifts. However, most of these solutions are complex and non-printable, which restricts their scalability and ease of implementation.

In contrast, the proposed loop antenna sensor presents significant advantages. It is simple, printable, and compact (50 × 70 mm), providing a cost-effective and scalable solution for monitoring meat freshness through resonant frequency shifts. Despite its reduced size, the sensor maintains high sensitivity to dielectric changes, delivering accurate and reliable results.

This work bridges the gap between advanced sensing technologies and practical applications, positioning the proposed loop antenna sensor as an ideal candidate for real-time food quality monitoring in supply chains to ensure freshness and minimize waste.

## 5. Conclusions

This study presents a flexible loop antenna-based sensor designed for real-time monitoring of meat freshness through dielectric property changes over a six-day period. The sensor demonstrated a sensitivity of 0.173GHz/day, with the resonance frequency shifting from 2.14 GHz to 1.29 GHz as the meat degraded. However, beyond Day 6, no significant frequency shifts were observed, indicating that the sensor’s detection limit for spoilage assessment had been reached. The experimental results aligned closely with simulations, showing minimal errors (maximum 0.04 GHz) and low standard deviations, confirming the system’s accuracy and repeatability.

While the sensor is compact (50 mm × 70 mm), printable, and cost-effective, it is most effective for monitoring thin meat cuts or surface-level freshness due to the need for the meat to be within the electromagnetic (EM) field to detect changes. This limits the sensor’s current application for larger meat portions and requires further research into multi-sensor or extended-range solutions to address these limitations.

Despite these constraints, the sensor holds significant potential for scalable food quality monitoring applications. However, since this is a proof-of-concept study, the sensor is intended to provide a relative, rather than absolute, assessment of freshness.

Future work may involve integrating the sensor into IoT platforms for automated real-time data collection, as well as exploring its application for other perishable foods and environmental factors. These developments could advance RF-based food safety solutions, offering an affordable and efficient tool to monitor food quality in various contexts.

## Figures and Tables

**Table 1:** Geometrical parameters of the proposed sensor.

Parameter	Value (mm)	Description
$WS_{L}$	50	Width of the substrate
$LS_{L}$	60	Length of the substrate
$W_{L}$	3	Width of the loop
$R_{L}$	20	Radius of the loop
$W_{f}$	10	Width of the feedline
$g_{L}$	4	Gap between loop and feedline
$L_{f}$	13	Length of the feedline
$h_{Loop}$	0.19	Substrate thickness
*t*	0.008	Conductor thickness

**Table 2:** Summary of simulated and measured resonant frequencies.

Day	$\epsilon_{r}$	Simulated Frequency (GHz)	Measured Avg Frequency (GHz)	Std. Dev (σ) (GHz)	Error (GHz)
0	37	2.10	2.14	0.022	0.04
1	40	2.05	2.05	0.025	0.00
2	45	1.90	1.89	0.014	0.01
3	57	1.70	1.72	0.022	0.02
4	74	1.50	1.51	0.048	0.01
5	91	1.30	1.29	0.038	0.01

**Table 3:** Overview of sensor types and their characteristics.

Ref.	Sensor Type	Detection Method	Samples	Measuring Parameters	Size (mm)	Fabrication Complexity
[19]	Collinear antenna	RF energy harvesting	Pork	RGB ^1^ color values	149 × 36	Simple/ Not-printable
[20]	Microwave antenna sensor	Dielectric characterization	Duck	Frequency shift	60 × 60	Simple/ Not-printable
[21]	Colorimetric sensor array	Detection of VOCs ^2^	Beef, chicken, fish, pork, shrimp	Concentration of VOCs ^2^ related to spoilage	787.4 × 279.4	Complex/ Not-printable
[14]	RFID ^3^ Tag antenna	RSSI ^4^ data	Frozen meat	Temperature	98 × 27	Complex/ Not-printable
[22]	Patch antenna	Dielectric characterization	Meat curing	Frequency shift	80 × 80	Simple/ Not-printable
[23]	Smart sensor tag	RF energy harvesting	Pork	Temperature, humidity	57.62 × 78.93	Complex/ Not-printable
This work	Loop antenna	Dielectric properties	Meat	Frequency shift	50 × 70	Simple/ Printable

^1^ Red, Green, and Blue; ^2^ Volatile Organic Compounds; ^3^ Radio Frequency Identification; ^4^ Received Signal Strength Indicator.

## Data Availability

Data are unavailable due to privacy restrictions.
